# Eliciting the Functional Taxonomy from protein annotations and taxa

**DOI:** 10.1038/srep31971

**Published:** 2016-08-18

**Authors:** Marco Falda, Enrico Lavezzo, Paolo Fontana, Luca Bianco, Michele Berselli, Elide Formentin, Stefano Toppo

**Affiliations:** 1 Department of Molecular Medicine, University of Padova, Padova, 35131, Italy; 2 Istituto Agrario San Michele all’Adige Research and Innovation Centre, Foundation Edmund Mach, Trento, 38010, Italy; 3 Department of Biology, University of Padova, Padova, 35131, Italy

## Abstract

The advances of omics technologies have triggered the production of an enormous volume of data coming from thousands of species. Meanwhile, joint international efforts like the Gene Ontology (GO) consortium have worked to provide functional information for a vast amount of proteins. With these data available, we have developed FunTaxIS, a tool that is the first attempt to infer functional taxonomy (i.e. how functions are distributed over taxa) combining functional and taxonomic information. FunTaxIS is able to define a taxon specific functional space by exploiting annotation frequencies in order to establish if a function can or cannot be used to annotate a certain species. The tool generates constraints between GO terms and taxa and then propagates these relations over the taxonomic tree and the GO graph. Since these constraints nearly cover the whole taxonomy, it is possible to obtain the mapping of a function over the taxonomy. FunTaxIS can be used to make functional comparative analyses among taxa, to detect improper associations between taxa and functions, and to discover how functional knowledge is either distributed or missing. A benchmark test set based on six different model species has been devised to get useful insights on the generated taxonomic rules.

Omics data have contributed to extend our knowledge in many fields of research and among these endeavours the characterization of protein molecular functions is one of the most challenging and prolific. Over time, experimental studies conducted on model organisms have been accumulated and progressively expanded to other species also by means of automatic functional transfer techniques through computational methods. The continuously growing taxonomic classification of living organisms on one hand, and the vast accumulation of protein functions on the other, have made necessary to deal with the increasing complexity of data. Therefore, the scientific community has tried to build up standards to be followed, not only in the classification of species, but also in the definitions of molecular functions. For the latter, a widely adopted resource for both accessing existing knowledge and contributing to its expansion is provided by the Gene Ontology Consortium (GOC), which is a comprehensive repository of functional information that relies on the use of a structured vocabulary, the Gene Ontology (GO)^1^. Currently, the GO Annotation database (GOA) contains over 280,000,000 annotations for almost 400,000 different species or inferior ranks^2^, and these numbers are increasing at a very high rate due to the recent development of “omics” technologies and in silico methods for protein function characterization^3^,^4^,^5^,^6^. These annotations are produced in two ways: manually, as a slow but accurate process carried out by trained curators and based on direct experimental results^7^, or automatically, as a faster approach that employs bioinformatics tools with minimal supervision^8^. Given the numbers involved, manual curation is not affordable for all gene products that continuously enter the public databases; as a consequence, the great majority of annotations originate from automatic pipelines (over 98%) and these annotations represent the only information available for many species. The quality of these automatically inferred annotations without any sort of supervision has been long debated but is increasing steadily and rivals the quality of the annotations checked by curators^9^.

The functions coded by the GO vocabulary are intended to be species-independent, but many of them represent functions, processes, and components that are not present in all kind of organisms, thus providing an implicit partitioning of the GO graph according to taxonomic criteria^10^,^11^. An example of such interconnections between functions and taxa is shown in Fig. 1: on the left there is a simplified view of the GO graph, which encodes functions with increasing levels of specificity from the root (“biological process” GO:0008150) to the leaves; on the right, the distribution of functions over the taxonomic tree is shown. In the example, the most specific term GO:0019878 (“lysine anabolism via aminoadipic acid”) refers to the peculiar biochemical pathway for the synthesis of the lysine that is used by fungi^12^ but never found in plants or mammals. Its ancestor, GO:0009085 (“lysine anabolism”), is more generic and forbidden only in mammals, which are not able to synthesize lysine. Finally, the two terms GO:0009067 (“aspartate family amino acid anabolism”) and GO:0006553 (“lysine metabolism”) are the less specific and they represent functions that can be used for all the taxa in the tree.

Even if the underlying principle of taxonomic partitioning of protein functions is accepted and easy to understand, its implementation is not trivial due to many factors, such as missing knowledge and errors in databases. The presence of inconsistencies between the taxon to which an annotated gene product belongs, and the implicit taxon specificity of the GO terms, was first raised in 2008 by Kusnierczyk^10^ and we noticed it while using Argot, our in-house developed tool for automated protein function prediction^15^.

The GOC started to deal with this problem back in 2010 and formalized an initial list of manually validated taxon-specific GO terms that is now freely available in the GO taxon constraints file. A tool was also created to detect and correct such inconsistencies^16^. Practically, GO taxon constraints are a list of statements that define the possibility to use or not a particular GO term to annotate a gene product of a given taxon. In particular, two types of relationships are used: “only_in_taxon” indicates that a GO term should be used to annotate only gene products originating from a given taxon or one of its descendants, while “never_in_taxon” forbids the use of a GO term for that taxon and its descendants. In addition, the concepts represented in the GO directed acyclic graph obey the “true path rule”: (i) a negative relationship extends to the child terms of the GO term to which it is referred, while (ii) a positive one is valid for all the parent GO terms.

The manual definition of constraints for almost 40,000 GO terms is not an affordable task and there is a strong need for an automated procedure as the one we have implemented in FunTaxIS (Functional Taxonomy Information System).

FunTaxIS is able to automatically generate GO taxon constraints, as illustrated in Fig. 2: starting from the protein level, it exploits GO annotations present in GOA and the frequencies of association between GO terms and taxa. Such constraints constitute the functional taxonomy, a sort of catalogue that describes taxa on the basis of the functions that they can or cannot perform. Notably, the constraints refer to taxa and not to specific proteins belonging to them: this means that a negative constraint for a particular taxon implies that none of its proteins should be able to perform such function, while a positive one suggests that at least one of them is able to achieve it.

FunTaxIS provides GO taxon constraints for most of the GO graph and is a freely accessible platform that can help:

- to detect, and then to correct, many improper associations between taxa and functions, especially among those automatically inferred (*e.g.* when using tools for function prediction);
- to complement phylogenomic studies^17^ or, more generally, to accomplish comparative analyses among taxa based on their functions;
- to visualize how functions are distributed among taxa;
- to compare taxa on the basis of their annotated functions;
- to put in evidence taxa that are unexpectedly weakly annotated and deserve to be further investigated;
- to reason over the evolutionary significance of gain and loss of functions.

Its effectiveness has been compared to the GOC constraints on simulated case studies of functional inference by sequence similarity, where the ability of the method to detect wrong annotations has been assessed.

A web application has been developed for accessing and visualizing the taxon constraints and is available at http://www.medcomp.medicina.unipd.it/funtaxis/.

## Methods

### Selection of GO terms

Only frequently occurring GO terms in GOA database have been considered. In particular, we did not include in the analyses the terms whose absolute frequency, cumulative over their descendants, was lower than 500. Though arbitrary, this cut-off has allowed us to generate a starting list of robust constraints that the propagation rules (explained in “Rules for propagating polarities in the taxonomic tree”) have then extended to the less frequent and previously not considered GO terms, thus partially recovering them.

### Selection of “general taxa”

The taxonomy database considered in this study (downloaded from NCBI^18^,^19^ on January 2, 2016) contains 1,117,863 nodes whose rank is equal or below the “species” level. We have reduced its complexity for the following reasons: (1) very few species are functionally characterized (only 1,401 different species or inferior ranks are associated with at least one experimental annotation, corresponding to 0.12% of total species), (2) closely related species generally possess very similar annotations; (3) specific GO terms, which are required to discriminate closely related species, are often present in GOA with a very low occurrence and cannot be used to generate reliable statistics.

Practically, only nodes ranked as “class” or above “class” in the NCBI taxonomy have been evaluated, and we will refer to them as “general taxa”. Furthermore, we have exploited other taxonomies, such as ITIS (Integrated Taxonomic Information System, retrieved on January 15, 2016, from http://www.itis.gov) and Wikispecies, to gather consensus rank information for nodes with missing information, collecting a total of 532 nodes. Once filled several “holes” in the ranks, we have mapped each taxon, whose rank is under “class”, to the most specific “general taxon”.

### Definition of robust general taxa

We have defined as “robust general taxa” only the highly annotated taxa that are able to provide sufficient information to support statistical inferences.

To quantify the degree of functional knowledge that exists for a certain general taxon, we have retrieved the proteins belonging to all taxonomic nodes subsumed by that general taxon, together with their GO terms. Then we have partitioned the taxonomic tree into seven big groups, according to the kingdom/superkingdom ranks (see Table 1); moreover, the Metazoa group has been further split into two additional groups, “Chordata” and “Metazoa excluding Chordata”, resulting in the scenario depicted in Supplementary Fig. S1.1. Within each group, a model organism has been identified and the other members of the group have been compared against it. To accomplish the task, the number of unique GO terms have been considered. A candidate general taxon is labelled as “robust” if it has more than 75% of unique annotations with respect to the amount of unique annotations of its reference model organism.

### Probability of association between GO terms and taxa

The robust general taxa, previously defined, have been tested against all GO terms in order to determine the relative probability of finding a certain GO term associated to a particular general taxon. Let *g* be a GO term belonging to the sub-ontology *A*, *c*_*t,g*_ the number of proteins of a given taxon *t* annotated with *g* or one of its children, *c*_*g*_ the number of proteins annotated with *g* or one of its children in the whole GOA, ^*A*^*c* the number of proteins in GOA annotated with at least a GO term whose sub-ontology is *A*, and ^*A*^*c*_*t*_ the number of proteins belonging to the general taxon *t* and annotated with at least a GO term whose sub-ontology is *A*. We can now define the relative probability as:


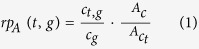


### Definition of GO taxon constraints

The relative probabilities are further transformed in the range −1 and +1, defining either negative or positive “polarities” respectively (*i.e.* “prohibition” or “possibility” of association, respectively; see Supplementary Section S2), while GO terms without a clear polarity with respect to a taxon are defined as “neutral”. Starting from these polarities, taxonomic constraints have been created: (i) a GO term with a positive polarity has been referred to as “in taxon”, meaning that it can be used to annotate the taxon itself and all of its children; (ii) a negative polarity is instead a “never in taxon” constraint, as neither that taxon nor any of its children can be annotated with that GO term. Note that, when there are no proteins linking a GO term to a general taxon, a dual strategy to decide how to classify that context has been adopted: if the taxon is robust (highly studied), a closed world assumption is made and then a “never in” constraint is inferred, otherwise an open world assumption is made^20^ and no constraints are applied (that is a “neutral” scenario). Exceptions are made when experimentally validated functions for a taxon are found: regardless of their relative probability, we let manually curated annotations prevail over inferences based on their frequency.

### Rules for propagating polarities in the taxonomic tree

**Figure 3:**
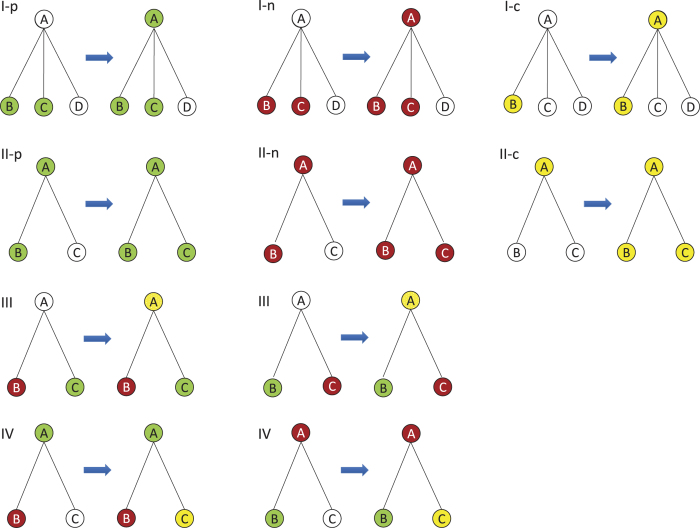
Propagation rules in the taxonomic tree. Red and green nodes represent taxa with negative and positive constraints, respectively. Yellow nodes are dubious. See “Methods” for extended details on the applied rules.

Polarities generated for robust general taxa are transferred to the other taxonomic nodes by applying a series of rules that are summarized in the following (see Fig. 3).

- Rule I: polar children propagate to the neutral parent, unless there are conflicts among siblings (see Rule III).
- Rule II: a polar parent propagates its polarity to all the neutral children, unless at least one of them has the opposite polarity (see Rule IV).
- Rule III: if two siblings have an opposite polarity, or one of them is ambiguous, their parent becomes ambiguous as well as their neutral siblings.
- Rule IV: if a polar parent has at least one child with the opposite polarity, all other neutral children become ambiguous.

These rules have been challenged with alternative propagation criteria and, compared to GOC constraints, showed better performance (see Supplementary Section S3).

### Rules for propagating polarities in the GO graph

A further strategy to expand the coverage of the GO constraints is the implementation of the “true path” rule which governs the GO graph. This rule says that if A is a parent of B then any property of A is inherited by B and, vice versa, if B is a child of A then any property not in A is also not in B. In logical terms:


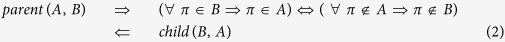


Two propagation rules follow:

1. positive polarities, or “in taxon” constraints, propagate toward the root;
2. negative polarities, or “never in taxon” constraints, propagate toward the leaves.

The true path rule has been applied to infer constraints for some of the GO terms that are not directly evaluated because too rare or even absent in the GOA; in particular, negative constraints can be propagated downwards to the leaves of the graph. This strategy has led to the inference of constraints for 13,781 GO terms that were not addressed in the first phase.

### Robustness analysis

The robustness of the method has been assessed by perturbing original annotations both in frequency and in absolute quantity and by comparing the results with the subset of manual constraints provided by GOC (Supplementary Section S3). Furthermore, in order to assess the statistical significance of the generated taxonomic constraints (*i.e* assignment of a p-value to these constraints) we have implemented a bootstrap approach (Supplementary Section S2).

### Benchmarking on case studies of proteome annotation

The potential benefit of the application of taxon constraints has been evaluated on simulated cases based on different model organisms, namely *S. cerevisiae*, *E. coli*, *A. thaliana*, *D. rerio*, *D. melanogaster*, and *H. sapiens*. Briefly, we have assessed how functions can be inherited by a sequence similarity driven approach based on BLAST^21^ searches *vs.* UniProt: for each query protein, the GO terms related to the first 100 BLAST hits have been retrieved from GOA, with e-values used as scores. To enrich the retrieval of GO terms from distant species, which may be functionally distinct from the model organisms and may carry incompatible GO annotations, we have removed from UniProt all the proteins of the corresponding taxonomic class (Saccharomycetes, Gammaproteobacteria, eudicotyledons, Actinopteri, Insecta, and Mammalia, respectively). Finally, the GO terms obtained with this method for the six proteomes have been filtered using taxon constraints: those provided by GOC, those generated by FunTaxIS, and their union have been applied independently, either with the open and the closed world hypotheses. In the first strategy, neutral cases are considered as “positive”, whereas in the second case they are rejected. The taxon compatibility of the results has been evaluated with a GO-centric approach: for each organism, the set of non redundant GO terms has been extracted from BLAST results (keeping the maximum score among all of the occurrences of each term) and has been compared with the set of non redundant GO terms associated to its proteins in GOA database, considered as the gold standard. Before the assessment, all the retrieved GO terms and those originally annotated on the benchmark proteins (true terms) have been propagated to the root of the ontologies.

## Results

FunTaxIS is a tool for the large scale generation of taxon constraints intended to define the range of functions that can be employed to annotate the gene products of a particular species. These constraints, coded as “in taxon” or “never in taxon”, are produced starting from the frequency of association between GO terms and taxa in the GOA database. The taxonomic constraints can be easily accessed and visualized at the following url: http://www.medcomp.medicina.unipd.it/funtaxis/.

### Taxonomic tree reduction

To simplify the taxonomic tree, all the species, genera, families, and orders have been collapsed into higher taxonomic levels, that we call “general taxa”, starting from the “class” rank: each taxon under the “class” rank has been associated with its nearest parent whose rank is equal or higher than “class”.

Since we observed that the large majority of these general taxa were poorly characterized from a functional point of view, we decided to partition the taxonomic tree and found, within each sub-part, some well-studied nodes that could serve as reference for poorly studied branches. The partitioning follows the kingdom/superkingdom taxonomic classification, with the exception of Metazoa that has been further divided into “Chordata” and “Metazoa excluding Chordata”: within each group, the general taxon with the highest number of unique GO terms has been selected as reference taxon. The other members of the groups have been compared with the corresponding reference taxa and those possessing an adequate number of unique annotations have been selected as “robust general taxa”. This final list is the starting point for the generation of the taxon constraints (Supplementary Section S1).

### GO terms evaluated

To quantify the impact of FunTaxIS on the whole GO, we computed the coverage of the GO terms addressed by at least one taxon constraint. For this purpose, the propagation rules described in the “Methods” section have been applied; for comparison, the coverage obtained by GOC constraints was also computed. The results, reported in Table 2, show that FunTaxIS is able to infer taxon constraints on a wider subset of functions. Interestingly, the relative gain is bigger for the Molecular Function ontology, where only 74 terms are addressed by constraints provided by GOC.

### Generation of all taxonomic constraints and comparison with the existing constraints from the GO Consortium

Since a taxonomic constraint can be either positive or negative, namely a GO term can be annotated “in” a particular taxon and “never in” a different taxon, the maximum number of taxonomic constraints that can be generated is the number of considered taxa multiplied by the total number of GO terms. However, constraints can be aggregated in two different ways: (1) by assigning them to shallow taxa (*e.g.* a “never in” applied to Fungi is also valid for all its children taxa) and (2) by collapsing GO terms that have parent-child relationships (*e.g.* for the “true path rule” of the GO graph, a “never in” for a certain GO term must be applied to all its children, while an “in” is also valid for all its parents).

For an exhaustive assessment of FunTaxIS, we evaluated and compared the expanded version of constraints coming from GOC with those produced by FunTaxIS. In particular, the propagation rules described in “Methods” have been applied to extend constraints in the GO graph and to the taxonomic tree (but limited to the 532 general taxa of the reduced taxonomic tree), respectively.

Following these principles, a total of 11,396,196 constraints have been obtained. The two sets of constraints produced by GOC and FunTaxIS are largely in agreement (only the 0.1% of them is discordant): in particular, 18.84% of total constraints are predicted by both FunTaxIS and GOC, 5.09% is only present in GOC, while the remaining 75.97% is a completely new set provided exclusively by FunTaxIS.

FunTaxIS constraints were also compared against the data provided by Peng and co-authors^22^ regarding human and yeast species, and the results show a very good agreement by the two methods. Details about these benchmarking analyses are reported in Supplementary Section S4.

### Graphical representation of functions

Once the taxonomic constraints have been generated, they can be projected either on the taxonomy, given a GO term, or on the GO graph, given a taxon. These orthogonal visualizations are both available in the FunTaxIS web server, in the “GO constraints” and “FunTasMaps” sections respectively, and are the essence of the functional taxonomy: the former allows the user to visually inspect the distribution of a certain function among taxa, the latter is the representation of the functional potentiality of a species and enables functional comparisons between different taxa. In the following, we will show a driven example for each of the approaches.

### GO-centric visualization

In the “GO constraints” section of the web server, the user is required to input a GO term in order to generate the complete list of constraints for all the taxa. An auto-complete function helps the user when inserting the GO term, providing proper suggestions when either a term name or a term ID are entered, while the drop down menu allows to select the type of constraints that will be shown, between those generated by FunTaxIS, those provided by GOC, and a prioritized combination of the two (GOC prevails). After clicking the “submit” button the results page is shown, divided into two sections: a representation of the taxonomic tree is shown at the top of the page for a visual inspection of the generated constraints, where the polarity is color-coded (green stands for “in taxon” constraint, red for “never in taxon”, blue/cyan for “neutral”, and yellow for “dubious”). Below the picture, a table reports the complete list of all the taxon constraints generated for the GO term inserted by the user, together with additional data regarding the number of proteins involved in its calculation and the origin of the constraints that can be inherited by either GO or taxon propagation rules. The whole dataset can be downloaded in tabular format to ease the implementation in downstream analyses and an extended “Examples” section is available in the web server.

A simple case can be explored by typing “GO:0007399” (“Nervous system development”). The GOC provides some constraints to limit the usage of this term, which are inherited from the parent term “multicellular organismal process” (GO:0032501). In particular, GOC reports that GO:0032501 should be used:

- “only in” taxon Eukaryota, meaning that this term and all of its children can be employed within this group but are banned for taxa outside Eukaryota;
- “never in” taxa *S. cerevisiae* and *S. pombe*, meaning that this term and all of its children cannot be used to annotate proteins belonging to these two species.

**Figure 4:**
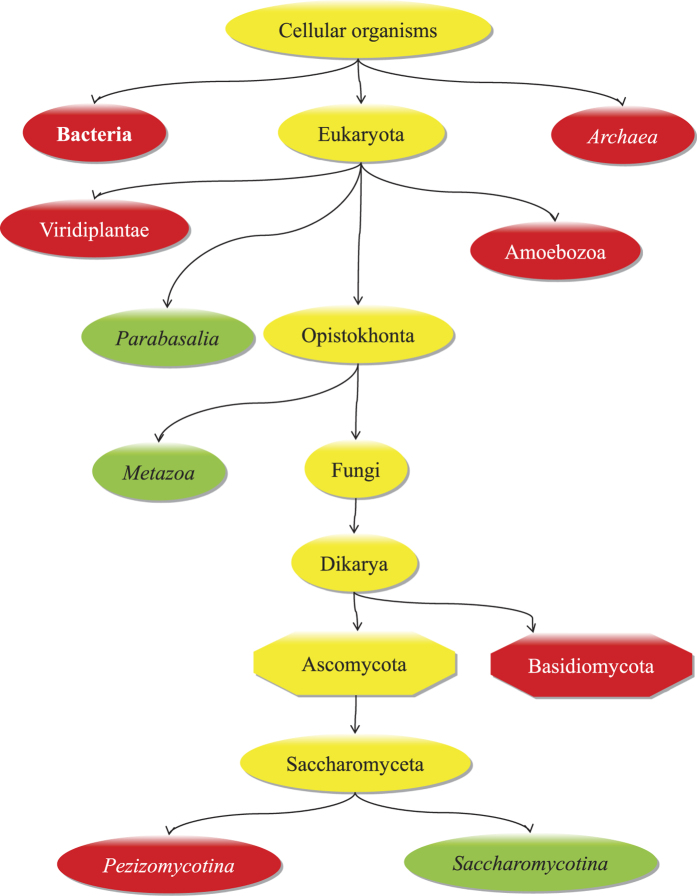
Simplified taxonomic tree produced by FunTaxIS for the GO term GO:0007399 (nervous system development). Nodes represent taxa, while the color code denotes the taxon constraints: red nodes are “never in” constraints, green nodes are “in”, yellow nodes are dubious (no decisions have been made).

Such constraints can be displayed by selecting “GO consortium” in the “Constraints” drop down menu. Interestingly, all taxa within Eukaryota, with the exception of *S. cerevisiae* and *S. pombe*, do not possess any explicitly negative constraint for the GO term GO:0007399; hence, in principle, they could be annotated with this term. Such nodes are all drawn in blue and marked as “neutral”, since the positive constraint provided by GOC is referred to a parent GO term and does not propagate downward the ontology. The only explicit positive constraint, expressly marked as a green node, regards the taxon Metazoa and is inherited by GO:0010001 (glial cell differentiation). The latter is a child node of GO:0007399, thus can be propagated upward the ontology. Similarly, the user can obtain the constraints inferred by FunTaxIS by simply changing the appropriate parameter from the “Constraints” drop down menu. An insight of the proposed taxonomic tree is shown in Fig. 4 starting from the root of the tree and going towards the leaves, we can observe that Archaea and Bacteria are labelled with a negative constraint (red nodes), in accordance with GOC, while Eukaryota is dubious (yellow) because it has child nodes with either positive and negative polarities. This is exactly where FunTaxIS can help to improve the state of the art: within the subtree subsumed by Eukaryota, it is able to produce a more fine-grained set of constraints, which can increase their discriminatory power and expand their range of applications. For example, additional negative labels are placed on Viridiplantae, Basidiomycota, Pezizomycotina, and Amoebozoa, while the taxa with an explicit positive association (green) are Metazoa, Saccharomycotina and Parabasalia. All the remaining nodes are dubious, since FunTaxIS was not able to assign any constraint. Obviously, the system is not always correct, as wrong constraints can sometimes arise, especially for poorly studied taxa or in the presence of errors in GOA database. As an example, GO:0007399 is allowed for Saccharomycotina: this inconsistency is due to the presence, in GOA, of a manual annotation associated to *C. albicans* (Vanadate, VAN1, resistance protein: UniProt accession Q00314), a species belonging to the Saccharomycotina subphylum. There is also the wrong positive constraint of the protists Parabasalia which is due to spurious electronically inferred annotations that should be amended. The capability of easily presenting constraints over taxa is a unique feature of FunTaxIS web interface that can help users to discover potential evident issues in the annotations and to take the correct actions.

### Taxon-centric visualization and comparison between taxa

We exploited the taxonomic constraints provided by FunTaxIS to build, for each most specific general taxon, a map of all its functions, accessible in the FunTasMaps section of the FunTaxIS web server. Such maps, that can be considered as functional fingerprints of taxa, are obtained by representing the GO graph in a circular space, where the colour code of nodes indicates functions that are present (green) or not present (red) in a given organism. To simplify the layout, functionally homogeneous GO terms with concordant taxonomic constraints can be collapsed into more generic functions: in this clustered view, nodes radii are proportional to the number of collapsed terms or supporting annotations.

**Figure 5:**
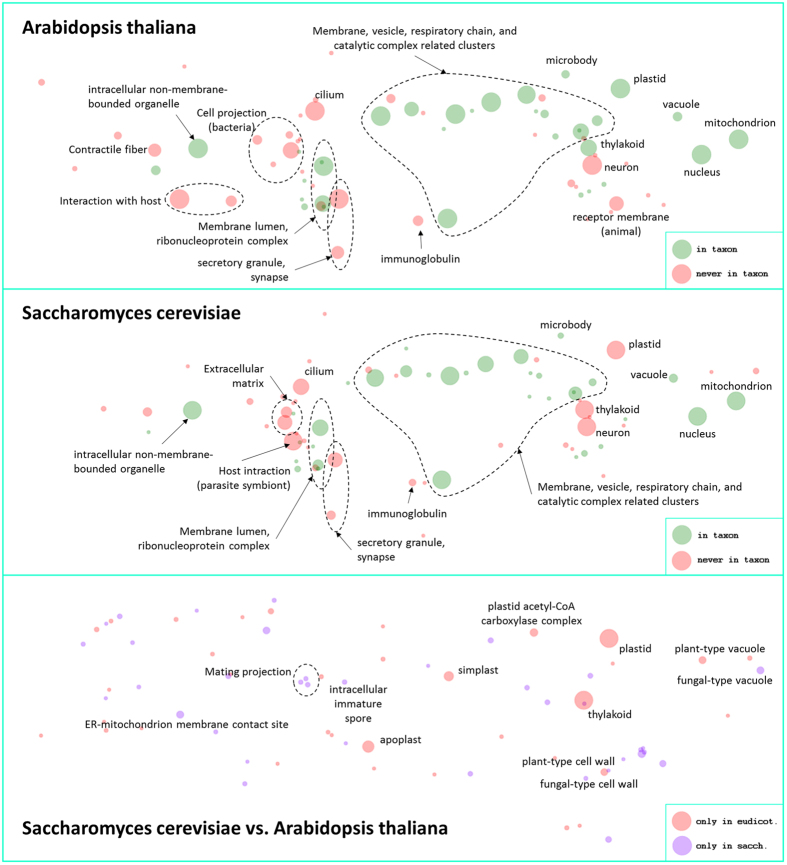
FunTasMaps of clustered constraints from cellular components of *A. thaliana* and *S. cerevisiae*. In the upper and middle boxes, the *A. thaliana* and *S. cerevisiae* functional fingerprints are shown, with labels highlighting the more significant GO clusters. Green and red circles refer to positive (“in taxon”) and negative (“never in taxon”) GO constraints, respectively. The bottom box reports the comparison of the two maps and some of the most relevant differences found between the two taxa are labeled. Red and violet circles refer to GO terms that are only present in *A. thaliana* and *S. cerevisiae*, respectively. The radius of the circles depends on the number of collapsed GO nodes or supporting annotations.

Interestingly, two species can be compared on the basis of their functions by overlapping their functional fingerprints, a feature attainable by inserting two organisms in the FunTasMaps form. In Fig. 5 the comparison between *S. cerevisiae* and *A. thaliana* is shown. The example is based on the clustered constraints derived from the Cellular Component sub-ontology and the most significant constraints are labelled. The fixed layout on which these constraints are placed helps to orientate the user and find the right correspondences on the map while comparing the differences between the two taxa. A list of the detailed differences is shown in the bottom box. Among the main differences that come to the surface there are plastid and thylakoid, typical of plants (*A.thaliana* in the example), and spore, mating projection etc. that are typical of fungi (*S. cerevisiae* in the example). Finally, these maps are even useful to unveil missing information and trace inconsistencies, as in the case of unexpected differences that could emerge between taxa because of lack of annotation in the database.

### Application of taxonomic constraints to case studies of sequence similarity-based functional transfer

We carried out some case studies of functional annotation of different organisms to prove the effectiveness of taxonomic constraints: here we report the results for *S. cerevisiae* and *A. thaliana*, whereas other species are presented in Supplementary Section S5. To simulate a difficult scenario, such as the case of non-model species with no clear homologs in UniProt, we removed from the database the proteins belonging to any species within the same taxonomic class; then, we considered the annotations of the first 100 hits retrieved by a BLAST search and their log-transformed e-values scores.

**Figure 6:**
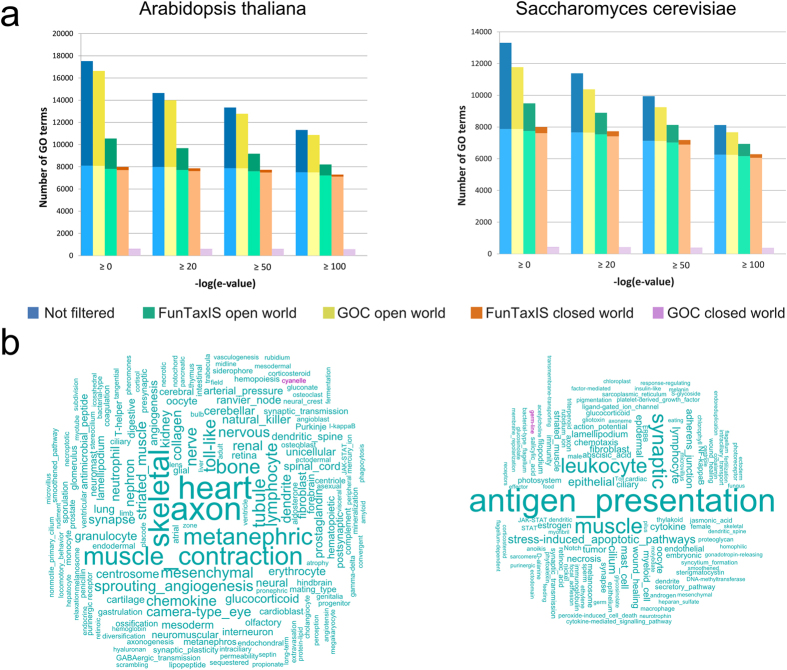
GO-centric evaluation of the impact of taxon constraints on *A. thaliana* and *S. cerevisiae* annotation by sequence-based functional transfer. (**a**) Non redundant GO terms retrieved by BLAST hits and grouped by the e-value of the alignment. The lower bright portion of columns represents true positives, that are the GO terms associated in GOA to at least one protein belonging to *A. thaliana* (left) or *S. cerevisiae* (right); the upper dark fraction represents false positives (GO terms never associated to *A. thaliana* or *S. cerevisiae* proteins). “Open” and “closed” world refer to the treatment of GO terms without any explicit constraint: such terms have been either discarded (closed) or retained (open); (**b**) Word clouds of the most frequent terms contained in GO definitions of false positive annotations (*A. thaliana* and *S. cerevisiae* respectively): turquoise and purple words come from GO terms with no defined constraints from GOC and FunTaxIS, respectively. The size of terms is proportional to their frequency (*e.g*. “muscle” is found in 74 different GO terms).

The annotations produced with this method have been processed by applying taxonomic constraints derived from GOC and from FunTaxIS. Two different strategies have been adopted for those GO terms that are “neutral”, *i.e.* they do not possess a defined constraint for the analysed species: in the “closed world” hypothesis such terms have been discarded from the results, while in the “open world” they have been kept. The GO-centric results of the benchmark are shown in Fig. 6: all non-redundant GO terms retrieved from BLAST hits are reported in the histograms (blue column), where bright and dark portions of bars represent true and false positives, respectively. The application of GOC (yellow) and FunTaxIS (bluish green) constraints results in a loss of GO terms, which prevalently affects those belonging to false positives. The higher coverage of FunTaxIS constraints has proven to outperform that provided by GOC and the benchmark highlights that FunTaxIS has been able to reduce a lot more “false positives”. The low coverage reached by GOC constraints is also the reason of the poor performance of “GOC constraints closed world”, since only GO terms with defined positive constraints survive this filtering step.

To give an idea of the different impact of GOC and FunTaxIS constraints, we computed the word clouds starting from the GO descriptions of false positive terms that survive taxonomic filters in the open world hypothesis. In the cloud, turquoise and purple words come from wrong GO terms that remain in the dataset after GOC and FunTaxIS filtering, respectively. Since the size of words correlate with their frequency, terms like “antigen presentation”, “muscle”, and “synaptic” are those more often found among false positives in yeast, and clearly belong to GO terms not compatible with such organism. The constraints provided by FunTaxIS are able to detect a wider range of potential inconsistencies compared to GOC, leading to the elimination of a bigger number of not appropriate annotations. It is important to note that wrong annotations do not originate exclusively from weak BLAST hits, but even when considering the GO terms coming from very significant BLAST hits (e-value < 1e-100, see Supplementary Section S6).

## Discussion

The functional characterization of genes and gene products has always played a central role in Biology, as it is the starting point for the elucidation of mechanisms and networks that govern the functioning of living organisms. The recent boost in omics sciences, particularly in the field of nucleic acids sequencing, has significantly increased the amount of data available for thousands of species, covering almost the whole taxonomy. Thanks to joint international efforts, the GO consortium has been able to create a rich vocabulary of functions, the Gene Ontology, consisting of terms that are organized in a graph structure and can be exploited to characterize protein sequences; such annotations are stored in the GOA database. Since each protein/gene stored in databanks usually contains information about the species of origin, we can obtain an implicit partitioning of the GO graph according to taxonomic criteria.

FunTaxIS has been designed to infer specificities and commonalities of the functions among different taxa. The idea is not completely new, since the GO consortium itself has recently introduced the concept of taxon constraint^16^, but the development of a method able to infer taxonomic constraints on large scale has not been proposed until now. FunTaxIS draws its conclusions starting from available information about the frequency of association between functions and taxa that can be retrieved from UniProt and GOA databases. The rationale is quite straightforward: if a certain function is often associated to proteins of a particular taxon, we can be somewhat confident that they are compatible. On the contrary, there are two possible explanations for the lack of association between a function and a taxon: (1) the function is not present in that species, and consequently should never be used to annotate its proteins, or (2) the taxon is poorly studied, so the lack of association does not guarantee that an incompatibility exists.

Based on these assumptions, FunTaxIS is able to infer the functional taxonomy, *i.e.* to determine which are the functions owned by a particular organism and those that are not or missing. The constraints are generated for nearly the whole taxonomy, exploiting information available for the most studied organisms to make inferences about the weakly characterized areas of the taxonomy.

The potentiality of FunTaxIS lies in the flexibility of its querying system that, together with the functional fingerprints that are available for all taxa, can be a valid starting point for further investigations about functional profiles, either shared or specific among taxa, and a good system to ease the detection of potential annotation errors in the database. As an example, we report a finding that surprised us at a first glance: GO term “cellular response to abscisic acid stimulus” (GO ID: GO:0071215) has a positive constraint for both Viridiplantae and Mammalia. Abscisic acid (ABA) is a well known plant hormone involved in many plant developmental processes^23^, but we were not aware that ABA has been also recently characterized in mammalian cells, where it regulates glucose homeostasis^24^.

Certainly, there are still issues that need to be addressed: positive constraints may originate from erroneous annotations in GOA, as in the case of Saccharomycotina for the term “Nervous system development”; or, on the contrary, some negative constraints may result from lack of annotations in certain taxa. But even in these cases, the output generated by FunTaxIS can be useful both for curators, in order to spot and then remove possible errors, and for biologists, that can be encouraged to investigate specific functions oddly absent in some taxa. In addition, the GOA is expected to grow significantly in the near future, both in terms of taxon coverage and annotation specificity; this supplementary data should allow, on the one hand, to refine the simplified version of the taxonomic tree adopted in this study by including deeper nodes, and on the other hand to expand the set of GO terms covered by taxonomic constraints.

Besides the usage discussed above, FunTaxIS can also be a valid support to address one of the issues concerning the annotation (assignment of GO terms) of the coding sequences from whole genome sequencing projects of non-model organisms. This part is usually carried out by tools for automatic protein function prediction that do not perform checks of annotation incompatibilities with the taxonomy of the target. As we directly observed, the major problems happen in the presence of difficult targets to predict, especially when there are no already characterized homologs and the sequence similarity approach is compromised. In such cases, it is easy for a predictive tool to take the wrong direction and deliver results that are not only wrong for the specific gene or protein, but are also completely incompatible for the species to which the gene/protein belongs. In this scenario, our automatic approach perfectly fits the need of detecting taxonomic inconsistencies, since it provides a high coverage of both the GO graph and the taxonomic tree, which is not affordable for manually curated constraints. We propose to integrate the constraints provided by FunTaxIS with those coming from the GO consortium, with the aim of improving both specificity and recall and obtaining a comprehensive system that could serve as guidance for the automatic function prediction. Negative constraints, in particular, are the key factors in this process, as they can be used to clean prediction results either by removing wrong annotations or by replacing them with more generic GO terms that are in the same path to the root but are not banned for the input species. The effectiveness of this approach has been already assessed in our automatic function prediction tool Argot2.5^15^ that has improved its performance thanks to the implementation of taxon constraints. The platform has been conceived to be constantly updated and, as a future development, we plan to enhance its precision by allowing users’ interaction and integrating their corrections.

## Additional Information

**How to cite this article**: Falda, M. *et al.* Eliciting the Functional Taxonomy from protein annotations and taxa. *Sci. Rep.* **6**, 31971; doi: 10.1038/srep31971 (2016).

## Supplementary Material

Supplementary Information

## Figures and Tables

**Figure 1:**
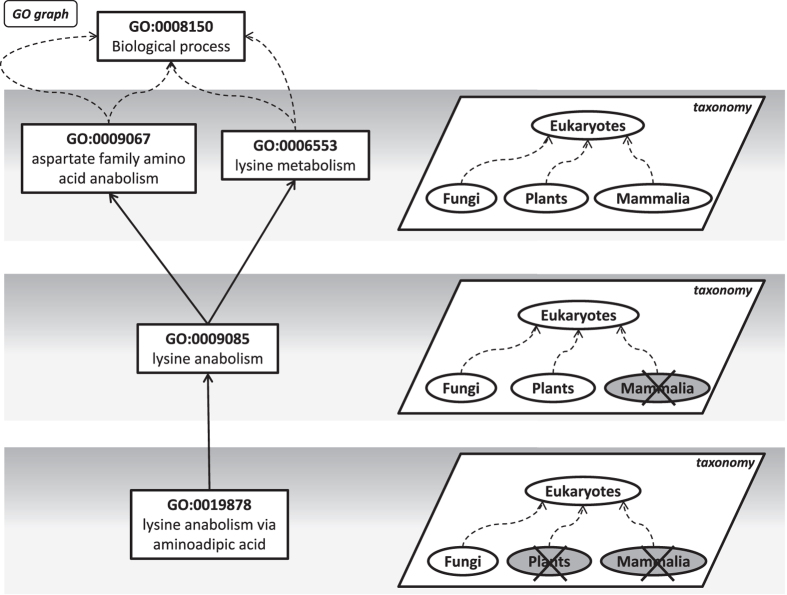
Distribution of functions over taxa. On the left, the GO graph represents functions with different levels of specificity, ranging from the most specific up to the most generic definitions. On the right, the application of the constraints on taxa: the term GO:0019878 (allowed for Fungi but denied for Plants and Mammalia, that are grey shaded) is more specific than GO:0009085 (allowed for Fungi and Plants), that in turn is more specific than GO:0009067 and GO:0006553 (both GO terms are allowed for Fungi, Plants and Mammalia). The root of the graph (GO:0008150) gathers all the biological processes.

**Figure 2:**
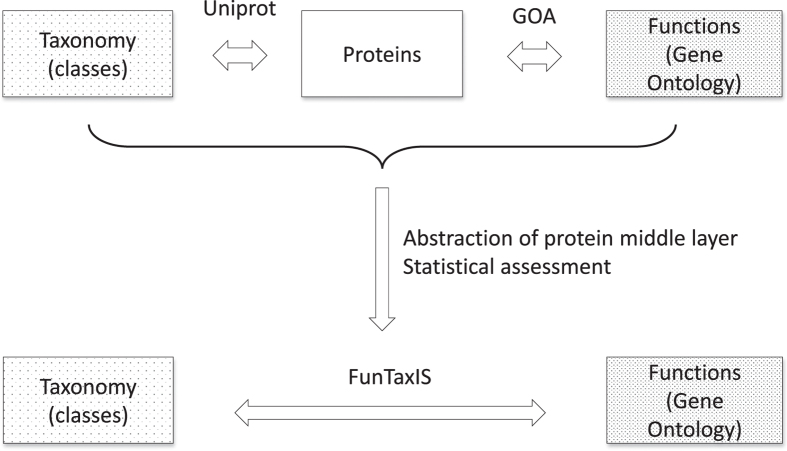
Working concept of FunTaxIS. FunTaxIS can take advantage of the existing links that relate proteins with GO functional annotations and the taxonomy of origin (upper part of figure). Through statistical considerations that allows both to assess and expand the functional attributions to species, our tool is able to abstract functions that are allowed or forbidden in some taxa directly linking functions to taxonomy (lower part of figure).

**Table 1:** Groups resulting from the partitioning of the taxonomic tree.

Groups		General taxon of the model organism		Model organism	
taxon ID	Groupsubsumer	taxon ID	General taxon name	taxonID	Species name
554915	Amoebozoa	142796	Mycetozoa	44689	*Dictyostelium discoideum*
2157	Archaea	183963	Halobacteria	2243	*Halobacterium sp.*
2	Bacteria	1236	Gammaproteobacteria	562	*Escherichia coli*
7711	Chordata	40674	Mammalia	9606	*Homo sapiens*
4751	Fungi	4891	Saccharomycetes	4932	*Saccharomyces cerevisiae*
33317	Metazoa excluding Chordata	50557	Insecta	7227	*Drosophila melanogaster*
33090	Viridiplantae	71240	Eudicotyledons	33090	*Arabidopsis thaliana*

For each group, the general taxon of the model organism and the model organism itself are reported. For Archaea, the most annotated organism has been selected.

**Table 2:** Coverage of taxon constraints on the whole set of GO terms.

	Biological Process	Molecular Function	Cellular Component	Total
GO terms	28,201	9,983	3,867	42,051
GO terms with at least 1 taxon constraint from FunTaxIS (%)	27,616 (97.93%)	8,584 (85.99%)	3,506 (90.66%)	39,707 (94.43%)
GO terms with at least 1 taxon constraint from GOC (%)	9,569 (33.93%)	74 (0.74%)	3,088 (79.86%)	12,731 (30.28%)
