# Supplementary material for: Eliciting the Functional Taxonomy from protein annotations and taxa
Source: Sci Rep. 2016 Aug 18;6:31971. doi: 10.1038/srep31971 (PMC4989186; doi:10.1038/srep31971)
Supplement: Supplementary Information [file srep31971-s1.pdf]

Eliciting the Functional Taxonomy from protein annotations and taxa

Marco Falda[1§], Enrico Lavezzo [1§], Paolo Fontana[2], Luca Bianco[2], Michele Berselli[1], Elide Formentin[3] and Stefano Toppo[1,*]

[1] Department of Molecular Medicine, University of Padova, Padova, 35131, Italy

[2] Istituto Agrario San Michele all'Adige Research and Innovation Centre, Foundation Edmund Mach, Trento, 38010, Italy

[3] Department of Biology, University of Padova, Padova, 35131, Italy

* To whom correspondence should be addressed. Tel: +39 049 8276958; Fax: +39 049 8073310; Email: stefano.toppo@unipd.it

[§] The authors wish it to be known that, in their opinion, the first two authors should be regarded as joint First Authors

SUPPORTING MATERIAL

Summary

SUPPORTING MATERIAL 2
S1. Taxonomy partitioning 3
S2. Fuzzy Logic and statistical tests........ 6
S3. Taxonomic propagation rules 8
S4. Benchmarking 14
S5. Additional case studies of the application of taxon constraints in sequence similarity-based functional transfer 16
S6. The origin of taxon-incompatible GO terms 21

S1. Taxonomy partitioning

In Figure S1.1, the partitioning of the taxonomic tree is shown. The tree has been divided into 7 groups, represented by the petals: within each group, the general taxa with the highest number of unique GO terms are shown in bold (with the most characterized species inside parentheses). In Table S1.1 all the highly annotated general taxa (robust general taxa) are reported.

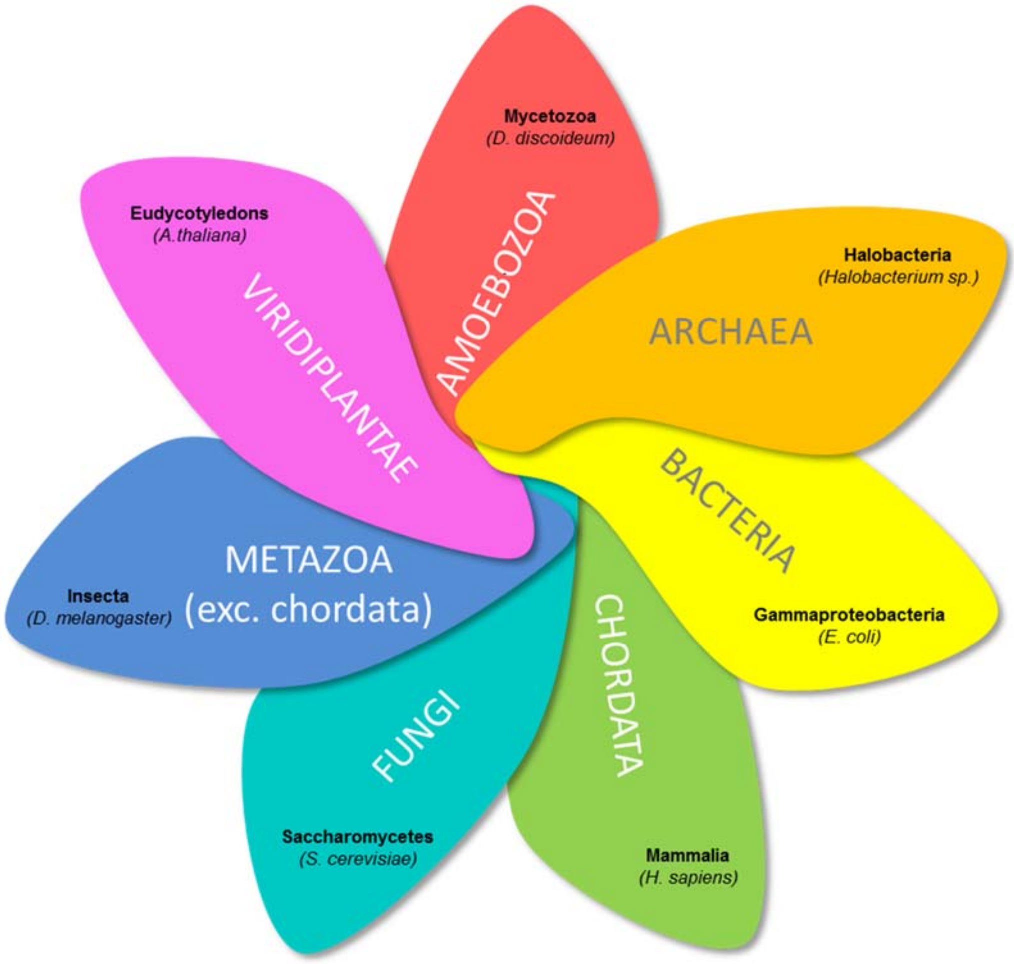

Figure S1.1: Taxonomy partitioning.

Table S1.1: robust taxa. General taxa selected as "robust" are reported, together with the taxonomic group they belong to, the number of non-redundant GO terms for the MF ontology used to annotate their proteins, the number of non-redundant GO terms for the MF ontology used to annotate the proteins of the corresponding reference taxon, and their ratio in percentage. The general taxa containing the reference organisms for each group are in bold.

Robust general taxon name	Group	N° of MF GO terms in the current general taxon	N° of MF GO terms in group reference taxon	% GO terms in current taxon *vs.* reference
Archamoebae	Amoebozoa	1190	1493	79.71%
Discosea	Amoebozoa	1154	1493	79.713%
Euglenozoa	Amoebozoa	1400	1493	93.77%
**Mycetozoa**	Amoebozoa	1493	1493	100%
Archaea	Archaea	1193	1552	76.87%
**Halobacteria**	Archaea	1552	1552	100%
Methanomicrobia	Archaea	1452	1552	93.56%
Thaumarchaeota	Archaea	13152	1552	84.73%
Thermoprotei	Archaea	1403	1552	90.40%
Actinobacteria	Bacteria	2281	2414	94.49%
Alphaproteobacteria	Bacteria	2242	2414	92.873%
Bacilli	Bacteria	22250	2414	92.17%
Bacteria	Bacteria	1864	2414	77.22%
Betaproteobacteria	Bacteria	2263	2414	93.74%
Clostridia	Bacteria	2103	2414	87.12%
Cyanobacteria	Bacteria	1982	2414	82.10%
Deltaproteobacteria	Bacteria	2080	2414	86.16%
Flavobacteriia	Bacteria	1863	2414	77.17%
**Gammaproteobacteria**	Bacteria	2414	2414	100%
Spirochaetia	Bacteria	1847	2414	76.51%
unclassified Bacteria	Bacteria	1924	2414	79.70%
Actinopteri	Chordata	1729	1942	89.03%
Amphibia	Chordata	1688	1942	86.92%
Ascidiacea	Chordata	1468	1942	
Aves	Chordata	1774	1942	91.35%
Cephalochordata	Chordata	1536	1942	
**Mammalia**	Chordata	1942	1942	100%
Testudines + Archosauria group	Chordata	1686	1942	86.82%
Agaricomycetes	Fungi	1496	1797	83.25%
Chytridiomycetes	Fungi	1397	1797	77.74%
Dothideomycetes	Fungi	1718	1797	95.60%
Eurotiomycetes	Fungi	1905	1797	106.01%
Leotiomycetes	Fungi	1656	1797	92.15%

Pezizomycetes	Fungi	1379	1797	76.74%
Pucciniomycotina	Fungi	1589	1797	88.43%
**Saccharomycetes**	Fungi	1797	1797	100%
Schizosaccharomycetes	Fungi	1542	1797	85.81%
Sordariomycetes	Fungi	1999	1797	111.24%
Tremellomycetes	Fungi	1493	1797	83.08%
Ustilaginomycetes	Fungi	1430	1797	79.58%
Arachnida	Metazoa excluding Chordata	1696	1970	86.09%
Chromadorea	Metazoa excluding Chordata	1698	1970	86.19%
**Insecta**	Metazoa excluding Chordata	1970	190	100%
Bryopsida	Viridiplantae	1543	2008	76.84%
Chlorophyceae	Viridiplantae	1679	2008	83.62%
**Eudicotyledons**	Viridiplantae	2008	2008	100%
Liliopsida	Viridiplantae	1735	2008	86.40%

S2. Fuzzy Logic and statistical tests

Relative probabilities have been defined to decide whether to consider a GO term *g* enough studied in a general taxon *t*, not enough represented or uncertain. Two thresholds ϑ_1 and ϑ_2, set to 0.1 and 1 respectively, seem to be enough discriminative, however, to lessen the precision on empirical values a smoothing around those thresholds using a trapezoidal preference function has been added. Besides the two thresholds ϑ_1 and ϑ_2, two uncertainty intervals δ_1 and δ_2, and two confidence intervals h_1 and h_2 around the estimated thresholds have been introduced. The resulting preference function can be designed as (see Figure S2.1):

$$fuzzy\_thr(x, \vartheta_1, \vartheta_2, \delta_1, \delta_2, h_1, h_2) = \begin{cases} -1 & if \quad x \le \vartheta_1 - \delta_1 \cdot (1 - h_1) \\ \dfrac{x - \vartheta_1 - \delta_1 \cdot h_1}{\delta_1} & if \quad x \le \vartheta_1 + \delta_1 \cdot h_1 \\ 0 & if \quad x \le \vartheta_2 - \delta_2 \cdot h_2 \\ \dfrac{x - \vartheta_2 + \delta_2 \cdot h_2}{\delta_2} & if \quad x \le \vartheta_2 + \delta_2 \cdot (1 - h_2) \\ 1 & otherwise \end{cases}$$

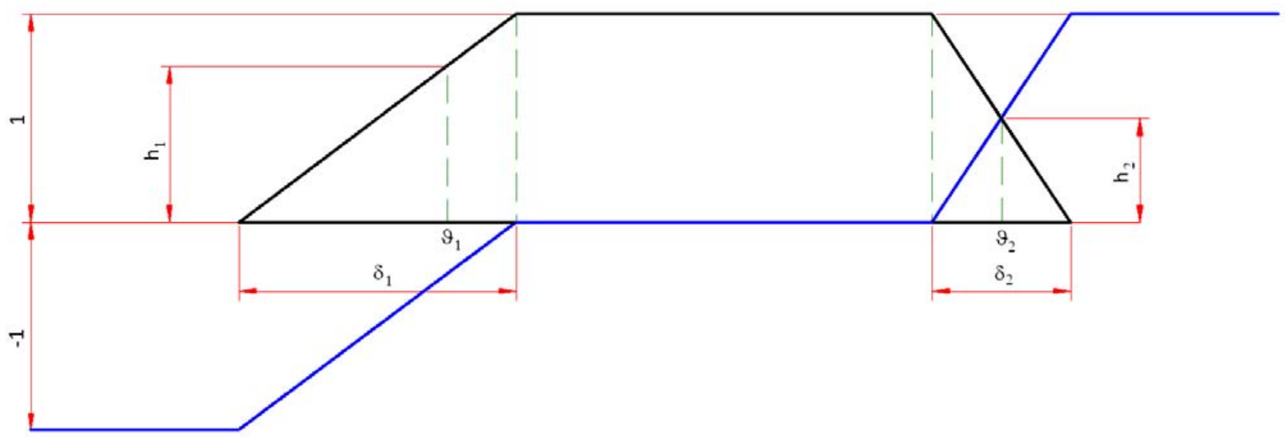

Figure S2.1: fuzzy threshold, in blue, and its generating trapezoid.

Fuzzy thresholds have been designed to obtain a more robust management of the uncertainty inherent the relative probabilities; moreover, by “abstracting” over the precise meanings of the relative probabilities thresholds, their precise semantics can be relaxed. Fuzzy thresholds likely improve the performances of the tool or, better, they improve the overall trade-off between performance and robustness. The current fuzzy thresholds are fairly stable: we run a Nelder-Mead optimization over the three preference function parameters ($h_1 = h_2 = 1$), that is the thresholds ϑ_1, ϑ_2, and $\delta_1 = \delta_2$; a 25-fold cross-validation has been performed over 63,965 initial GOC constraints, fitting vectors of 2,558 elements and obtaining the following optimized parameters:

- $\vartheta_1 = 0.11$ +/- 0.002 (standard deviation)
- $\vartheta_2 = 0.919$ +/- 0.189
- $\delta_1 = \delta 2 = 0.490$ +/- 0.089

In order to assess the statistical significance and assign a p-value to the taxonomic constraints, a bootstrap approach has been used since the data cannot be properly modeled with any known distribution. A one-sided t-hypothesis test has been adopted, and ‘in taxon’ constraints have been tested against the ‘never in taxon’ constraints distribution and vice versa. The test has been repeated for each constraint against 10,000 resampled distributions and the t scores obtained have been used to calculate the p-value considering a significance level of 5% (alpha <= 0.05). The p-values have finally been adjusted for multiple testing using the Bonferroni correction method.

S3. Taxonomic propagation rules

S3.1 Rules application

The frequency distribution of the taxonomic propagation rules is reported in Figure S3.1. The majority of constraints comes from propagations from children, which is expected since the algorithm follows a bottom-up strategy. Bottom-up propagations are almost equally shared among positive, negative and dubious children. The rarest are the dubious ones generated from a conflictual parent and a conflictual child. Figure S3.2 reports one example for each type of rule (see main text and Figure 2 therein); for example, in the case of rule I-p *Opisthokonta* acquire their polarity from *Echinoidea*, in rule II-p *Actinopteri* acquire the polarity indirectly from *Chondrichthyes* through *Craniata*. Dubious rules can originate from two general scenarios: Either when the polarity of the parent is discordant with respect to the polarity of a sibling (example IV; note that "*Eukaryota*" means "other *Eukaryota*", that are uncharacterized *Eukaryota*, see the web site), or when the polarities of two siblings are discordant (example III). Once a doubt appears, it taints all its neutral neighbors by propagating both upwards and downwards.

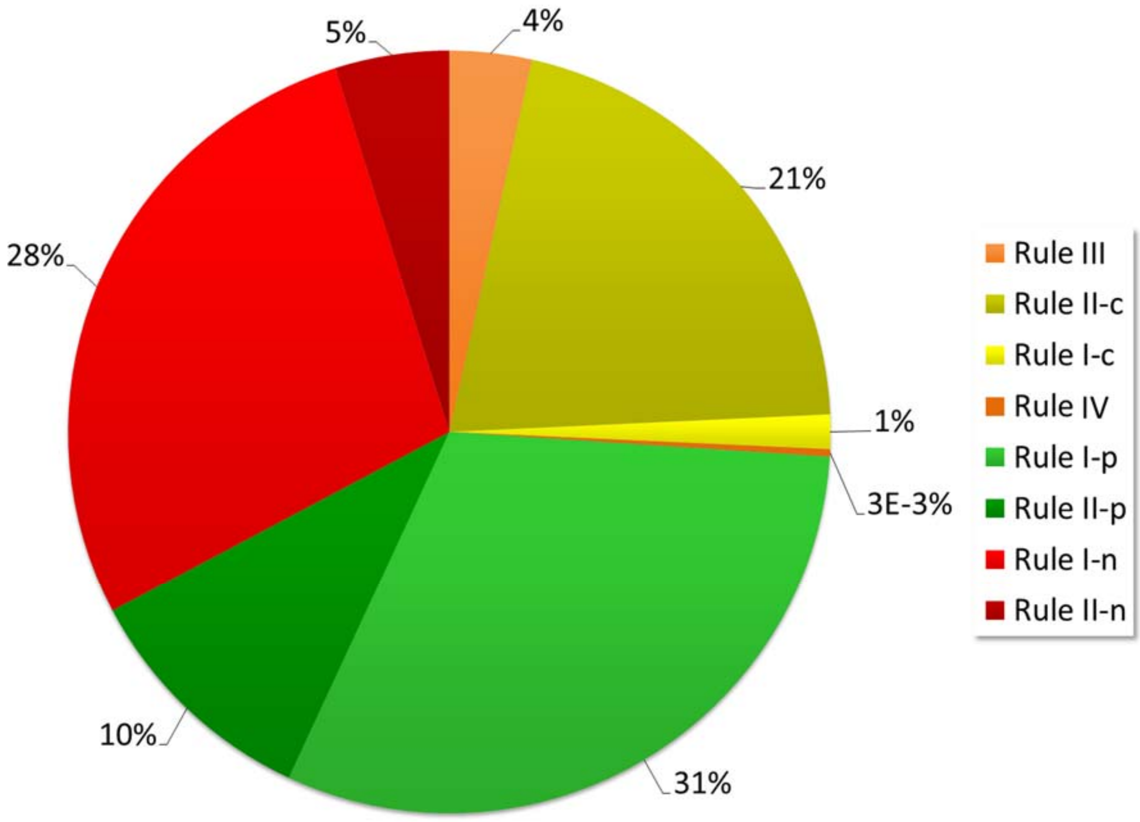

Figure S3.1: frequency distribution of the taxonomic propagation rules.

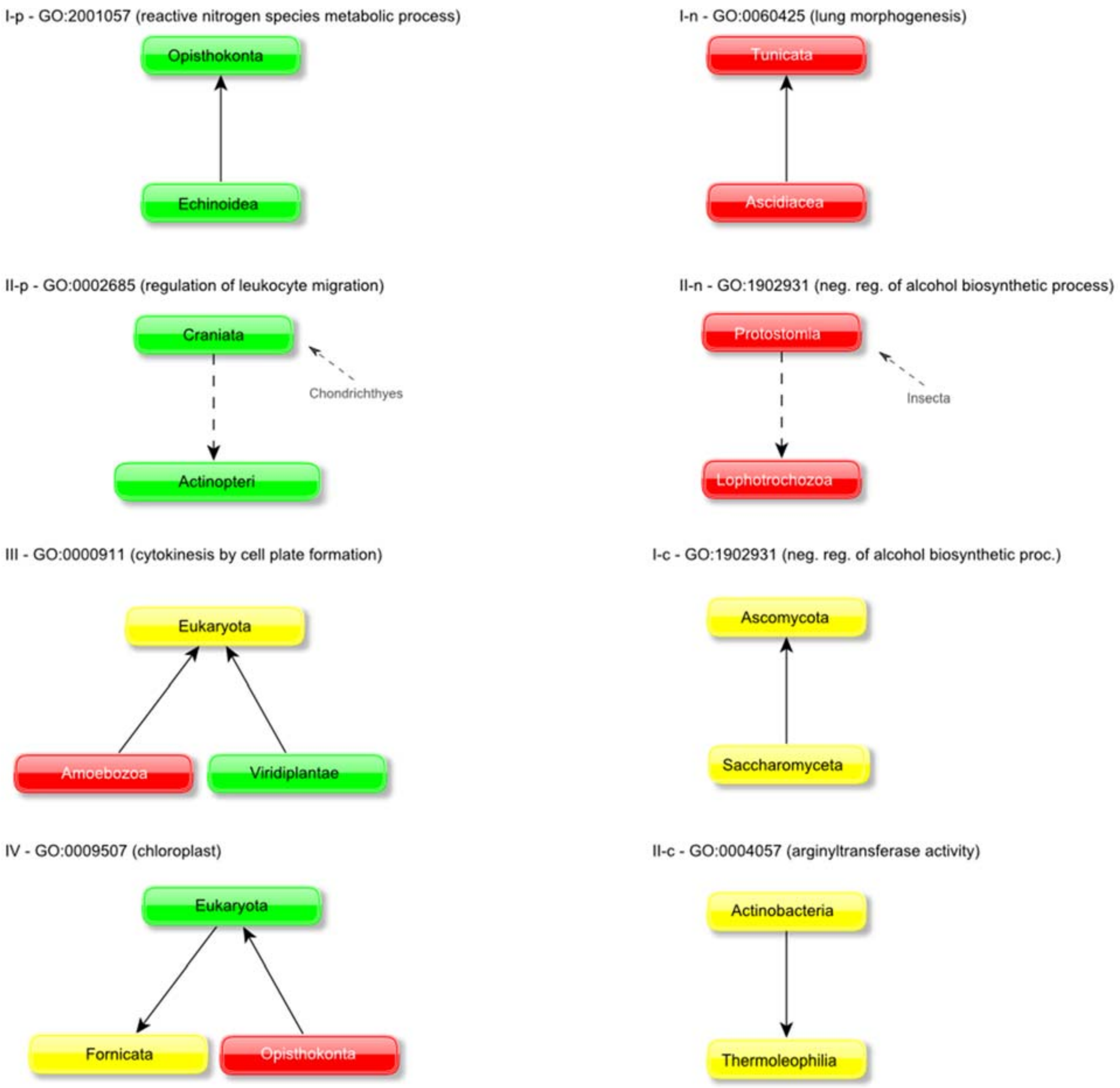

Figure S3.2: instances of taxonomic propagation rules.

S3.2 Arbitrariness and Robustness

To have an idea on how much arbitrary our taxonomic propagation rules are, two additional alternative propagation criteria have been designed: one based on an open world hypothesis and the other based on a closed world hypothesis (Figure S3.3). These rule sets have been then used to propagate the initial constraints over the Taxonomy tree and the results have been compared with the manual rules proposed by the GO Consortium (GOC in the following); results are shown in Figure S3.4 (the lower the better).

Another interesting point concerns the robustness of the generated rules; robustness can be defined as the resilience of the system when the input data, that is the protein annotations, are perturbed. Since a robust system far from optimality is not useful, first of all it has been established that the steady state deviation ss has to be measured with respect to the fraction of the common constraints between the FunTaxIS set $\mathcal{F}$ and the GOC set $\mathcal{G}$ whose resulting discretized polarity p_{set} is not the same

$$ss = \frac{\sum_{r \in \mathcal{F} \cap \mathcal{G}} \chi_{\{p_{\mathcal{F}}(r) = p_{\mathcal{G}}(r)\}}(r)}{\sum_{r \in \mathcal{F} \cap \mathcal{G}} \chi_{\mathcal{F} \cap \mathcal{G}}(r)}$$

where $\chi_S(x)$ is the characteristic function of a set S. In the case of a GOC constraint g, its polarity $p_{\mathcal{G}}(g)$ has been defined as

$$p_{\mathcal{G}}(g) = \begin{cases} -1, & g = \text{"}never\_in\text{"} \\ 1, & g = \text{"}only\_in\text{"} \end{cases}$$

The simulation is performed by applying the function $\pi_\delta : \mathbb{N}_+ \rightarrow \mathbb{N}_0$ to an increasingly wide random subsets α of annotations $\mathcal{A}$ by adding or subtracting a fixed amount δ of annotations according to a uniform Boolean random variable ϱ

$$\pi_\delta(x) = \begin{cases} x - \delta, & \varrho = \text{False} \\ x + \delta, & \varrho = \text{True} \end{cases}$$

Four random replicates have been produced in order to obtain also an approximate standard deviation. In Figure S3.5 the relative increments

$$\Delta_{ss_{\alpha,\delta}} = \frac{ss_\delta(\alpha) \cup ss_0(\mathcal{A} \backslash \alpha)}{ss_0}$$

of the steady state deviations produced by varying $\alpha \subseteq \mathcal{A}$ and δ, where $\mathcal{A}$ is the unperturbed set of annotations, have been plotted. It can be noticed that the wrong constraints increase along both axes and that the system tends to worsen for increasing numbers of perturbed annotations and for increasing amounts of noise annotations.

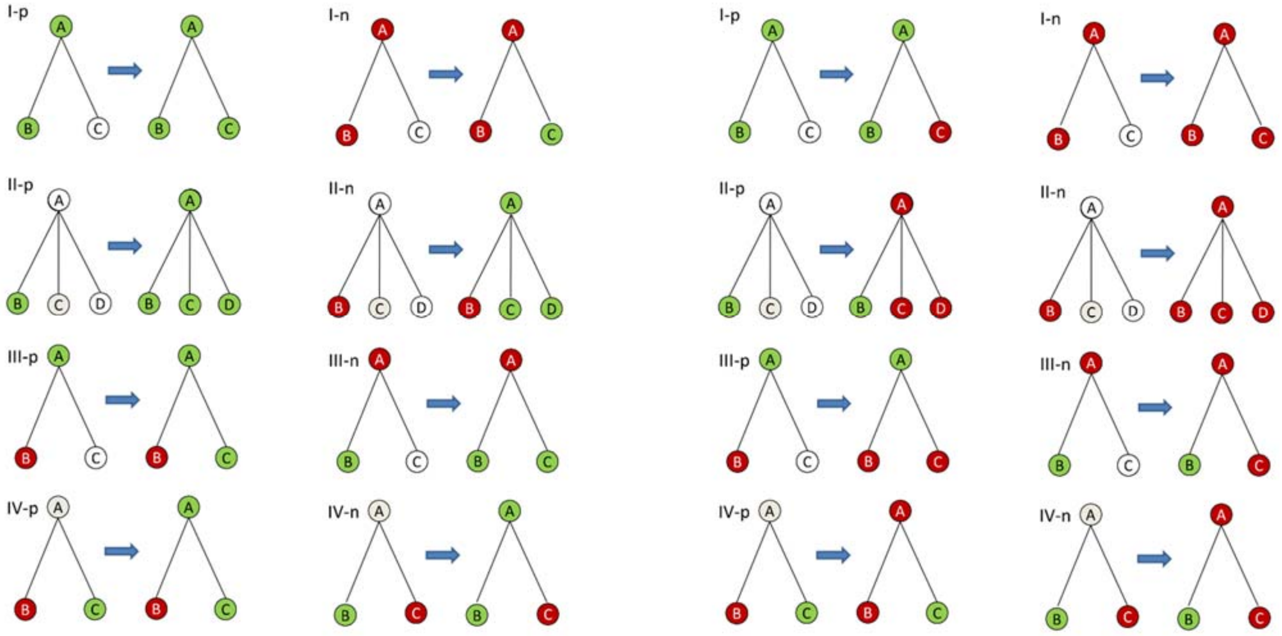

Figure S3.3: two additional alternative taxonomic propagation rules sets; on the left a set built from an open world hypothesis, while on the right a set built on a closed world hypothesis.

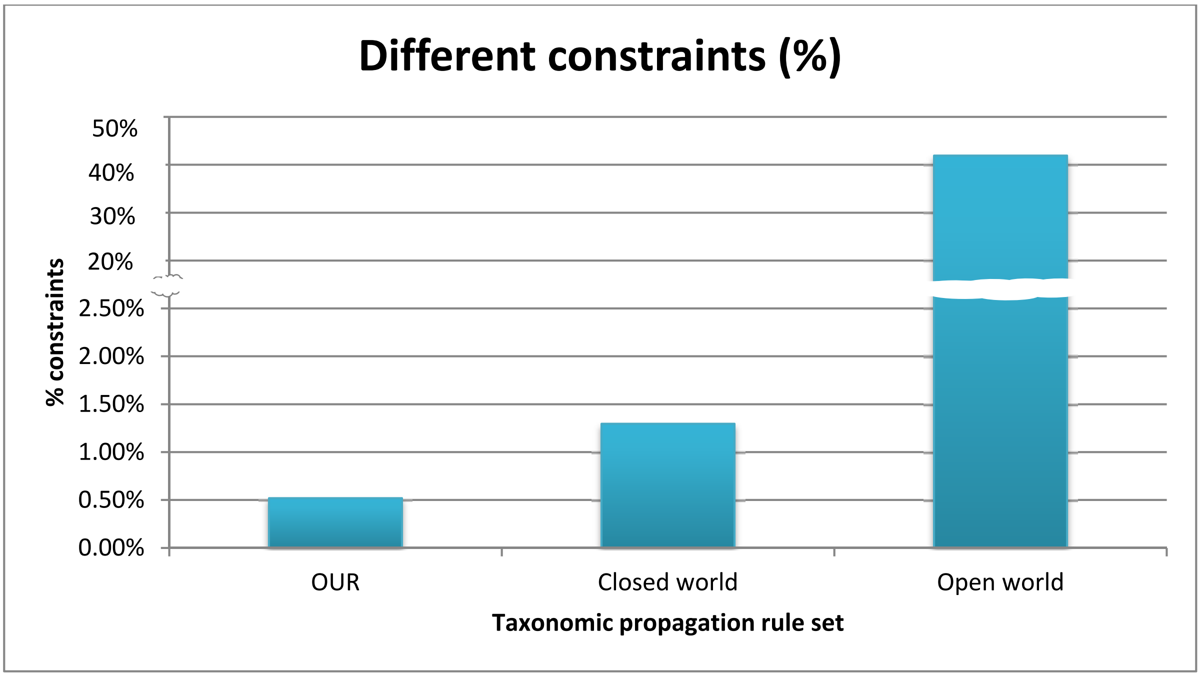

Figure S3.4: the number of wrong constraints, with respect to the GO Consortium, inferred by replacing the default taxonomic propagation rules (OUR) with one of the two alternative sets shown in Figure S3.3.

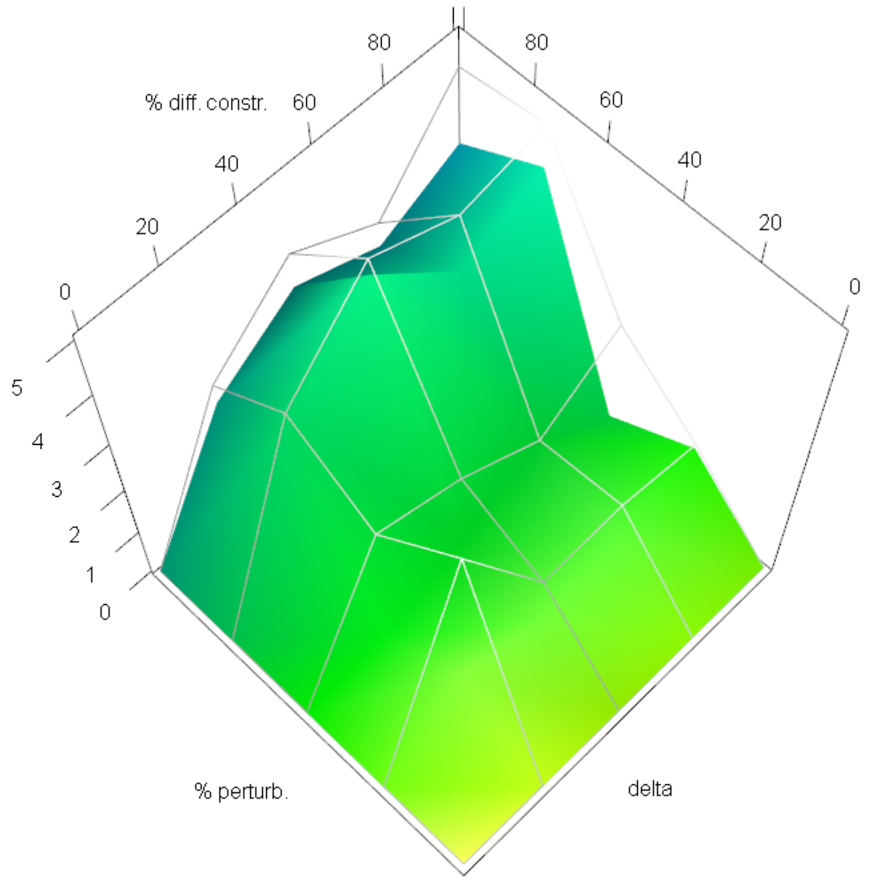

Figure S3.5: the relative variation of wrong constraints with respect to the percentage of wrong constraints estimated from the "true" GO Consortium dataset; this plot has been built by perturbing an increasing percentage of annotations (% perturb.) by a fixed increasing amount of noise annotations (delta). The grey grid over the surface gives an idea of the standard deviation coming from four random replicates. For "delta" equal 0, by increasing "% perturb" there is obviously no difference ("% diff constr." is 0) and the same happens for "% perturb" equal 0 and increasing "delta" as shown in the plot.

S3.3 Rules application and world hypotheses

The open or closed world hypotheses must be considered also when the constraints are to be applied to a set of GO terms. More precisely, a concrete decision on neutrality of terms must be taken, *i.e.* uncertain/neutral terms must be either accepted or rejected for the taxon. To simplify the reasoning, a sort of ternary logic setting can be adopted, where in addition to the canonical true, T, and false, ⊥, symbols there is a novel neutral symbol ⊣. The differences between the open and closed world hypotheses reduce to the intrepretation of the neutral symbol: tendentially true in the open world and tendentially false in the closed world. In dealing with two

possibly contradictory information sources, a fusion criterion has to be established. By giving a more authoritative role to the GOC source (Σ_G) with respect to FunTaxIS source (Σ_F), two slightly different truth tables (Table S3.1 and Table S3.2) can be devised for the open and closed world hypotheses respectively.

Table S3.1: truth table for the fusion of the GOC and FunTaxIS information sources in the hypotheses of open world and preferential bias towards the former source.

FunTaxIS decision (Σ_F)	GOC decision (Σ_G)	Final decision
⊥	⊥	⊥
⊥	⊣	⊥
⊥	T	T
⊣	⊥	⊥
⊣	⊣	T
⊣	T	T
T	⊥	⊥
T	⊣	T
T	T	T

Table S3.2: truth table for the fusion of the GOC and FunTaxIS information sources in the hypotheses of closed world and preferential bias towards the former source.

FunTaxIS decision (Σ_F)	GOC decision (Σ_G)	Final decision
⊥	⊥	⊥
⊥	⊣	⊥
⊥	T	T
⊣	⊥	⊥
⊣	⊣	⊥
⊣	T	T
T	⊥	⊥
T	⊣	T
T	T	T

From those truth tables it is possible to synthesize the following formulas for the two world hypotheses:

$$(\Sigma_G = \mathrm{T}) \vee (\Sigma_G = \dashv \wedge\, \Sigma_F \neq \perp)$$

$$(\Sigma_G = \mathrm{T}) \vee (\Sigma_G = \dashv \wedge\, \Sigma_F = \mathrm{T})$$

S4. Benchmarking

S4.1 Taxon constraints comparison between FunTaxIS and GOC

The Venn diagram in Figure S4.1 shows that taxon constraints provided by GOC largely overlap those generated by FunTaxIS, while numerical details are presented in Table S4.1. Only non-neutral constraints are represented, while there are additional 10,887 constraints (0.1% of the total) that are discordant between the two methods and are not reported in the chart.

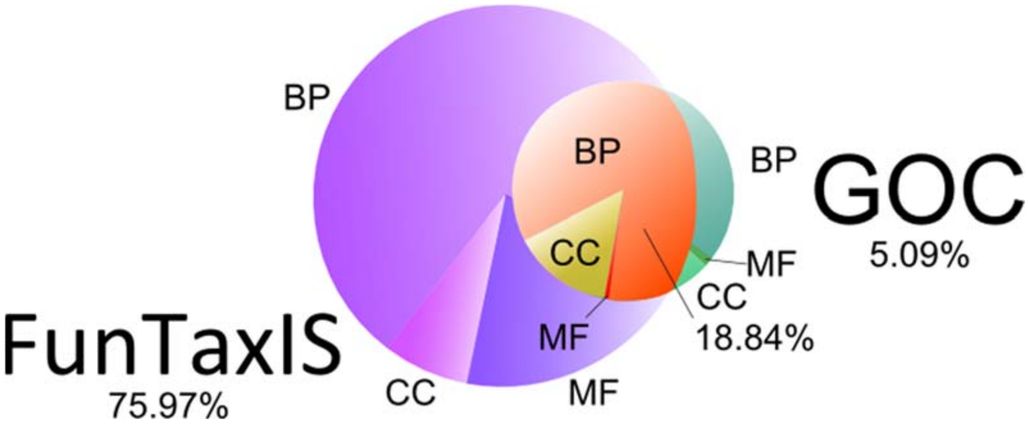

Figure S4.1: Overlap between GOC and FunTaxIS constraints.

Table S4.1: total number of non-neutral constraints in GOC and FunTaxIS.

Ontology	Constraint type	N° GOC constraints	N° FunTaxIS constraints
**BP**	Positive	162,459	1,389,199
	Negative	1,998,097	6,195,981
	Total	2,160,556	7,585,180
**MF**	Positive	4,147	776,112
	Negative	17,328	906,386
	Total	21,475	1,682,498
**CC**	Positive	62,809	252,985
	Negative	328,202	642,889
	Total	391,011	895,874
**Total**		2,573,042	10,163,552

S4.2 FunTaxIS vs CroGO

In a paper published in 2013 (1), the authors presented a tool able to estimate the similarity of GO terms from different ontologies. Together with the tool, they provided two species-specific lists (one for *S. cerevisiae* and one for *H. sapiens*) of coupled GO terms from the Molecular Function and the Biological Process ontologies characterized by high similarity. Since these terms are functions present in yeast and/or human, we exploited these data to perform an independent benchmark to assess the correctness of the constraints generated by FunTaxIS for those species (see results in Figure S4.2). In particular, the GO terms with a positive or neutral constraint in FunTaxIS were considered to be in agreement with CroGO (since we adopt the open world assumption), while those with a negative constraint were considered discordant (percentages are reported on top of the bars). The two datasets are largely in agreement, although being obtained with two completely different methods.

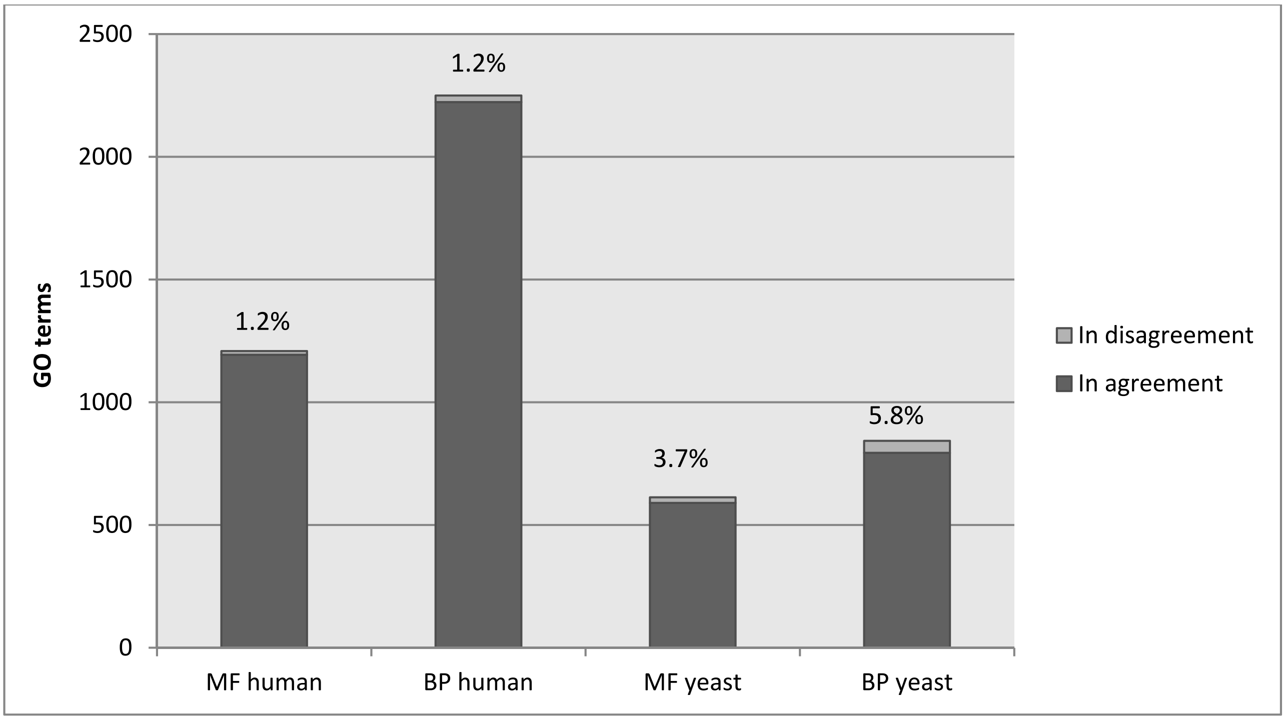

Figure S4.2: histogram reporting the percentage of agreement with CroGO dataset of true GO hits for human and yeast after been filtered by FunTaxIS constraints.

S5. Additional case studies of the application of taxon constraints in sequence similarity-based functional transfer

The effectiveness of taxonomic constraints has been tested in simulated cases of functional annotation based on sequence similarity. In addition to *S. cerevisiae* and *A. thaliana*, which are reported in the main text, we analysed *D. rerio*, *D. melanogaster*, *H.sapiens* and *E. coli*.

In the following figures, panels A are histograms of the frequencies of non redundant GO terms retrieved by BLAST hits and grouped by the e-value of the alignment. The lower bright portion of columns represents true positives, that are the GO terms associated in GOA to at least one protein belonging to the target species; the upper dark fraction represents false positives (GO terms never associated to the target species proteins). “Open” and “closed” world refer to the treatment of GO terms without an explicit constraint: such terms have been either discarded (closed) or retained (open). Panels B are word clouds of the most frequent terms contained in GO definitions of false positive annotations: turquois and purple words come from GO terms with no defined constraints from GOC and FunTaxIS, respectively. The size of terms is proportional to their frequency.

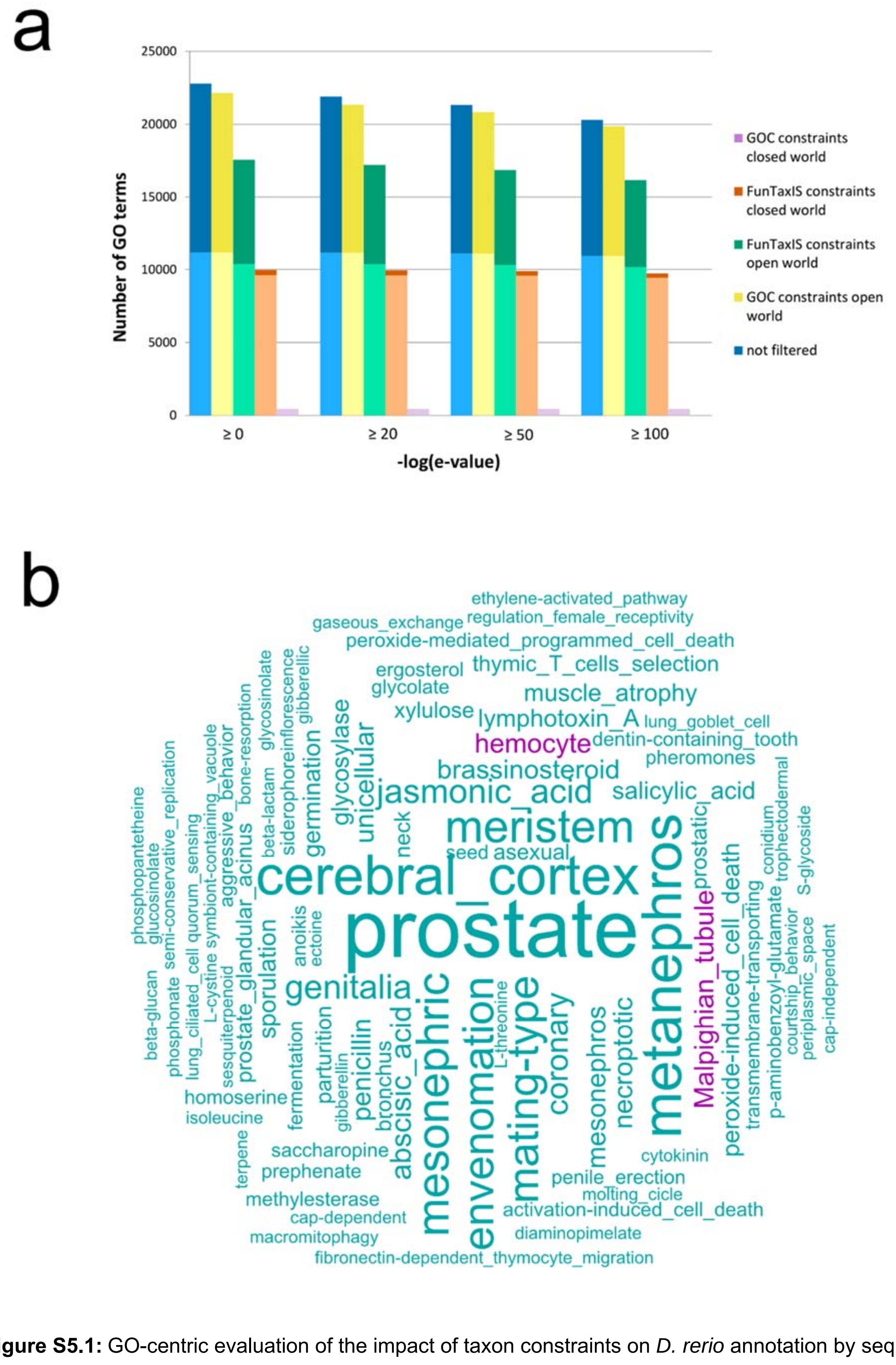

Figure S5.1: GO-centric evaluation of the impact of taxon constraints on *D. rerio* annotation by sequence-based functional transfer.

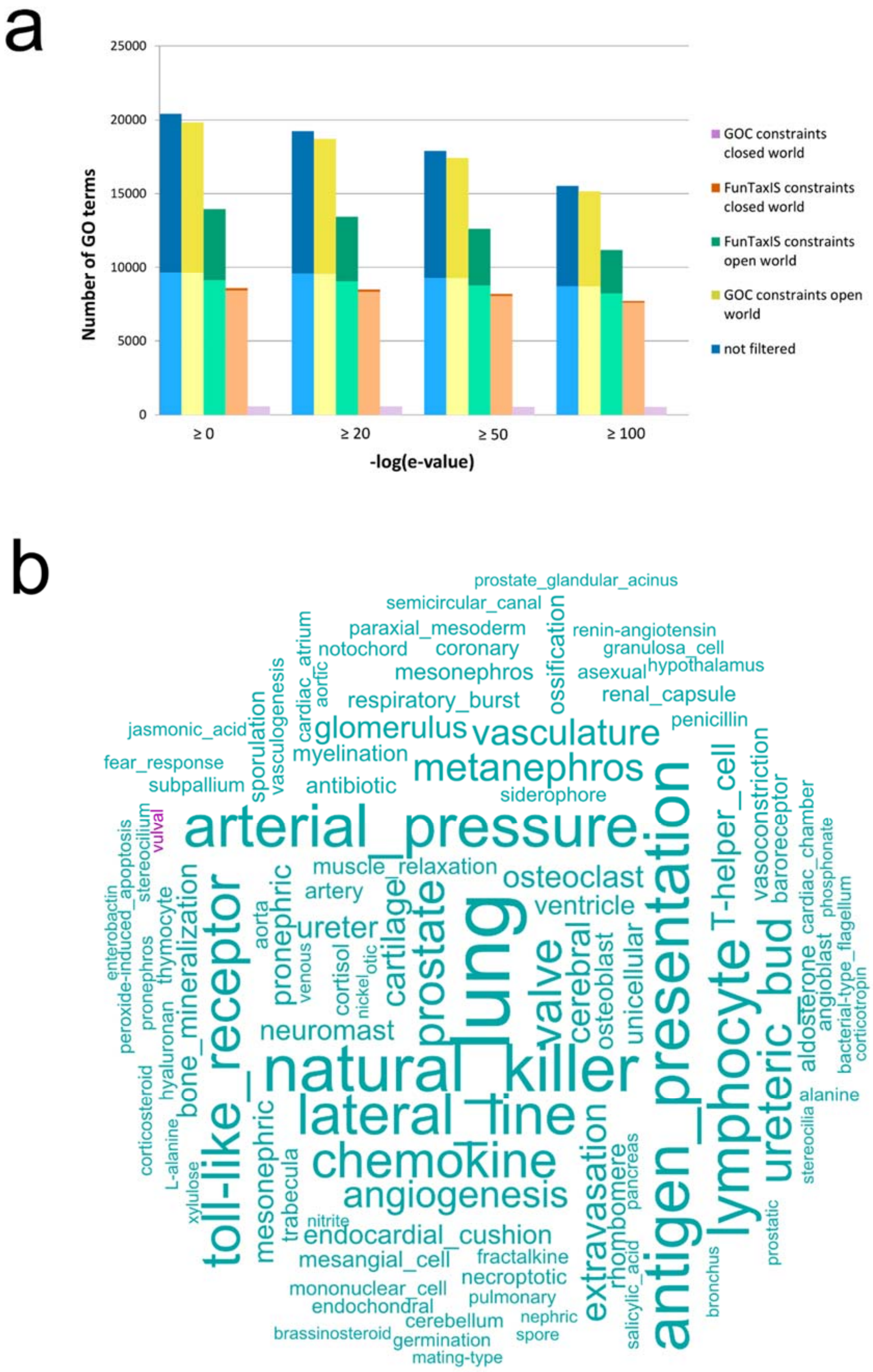

Figure S5.2: GO-centric evaluation of the impact of taxon constraints on *D. melanogaster* annotation by sequence-based functional transfer.

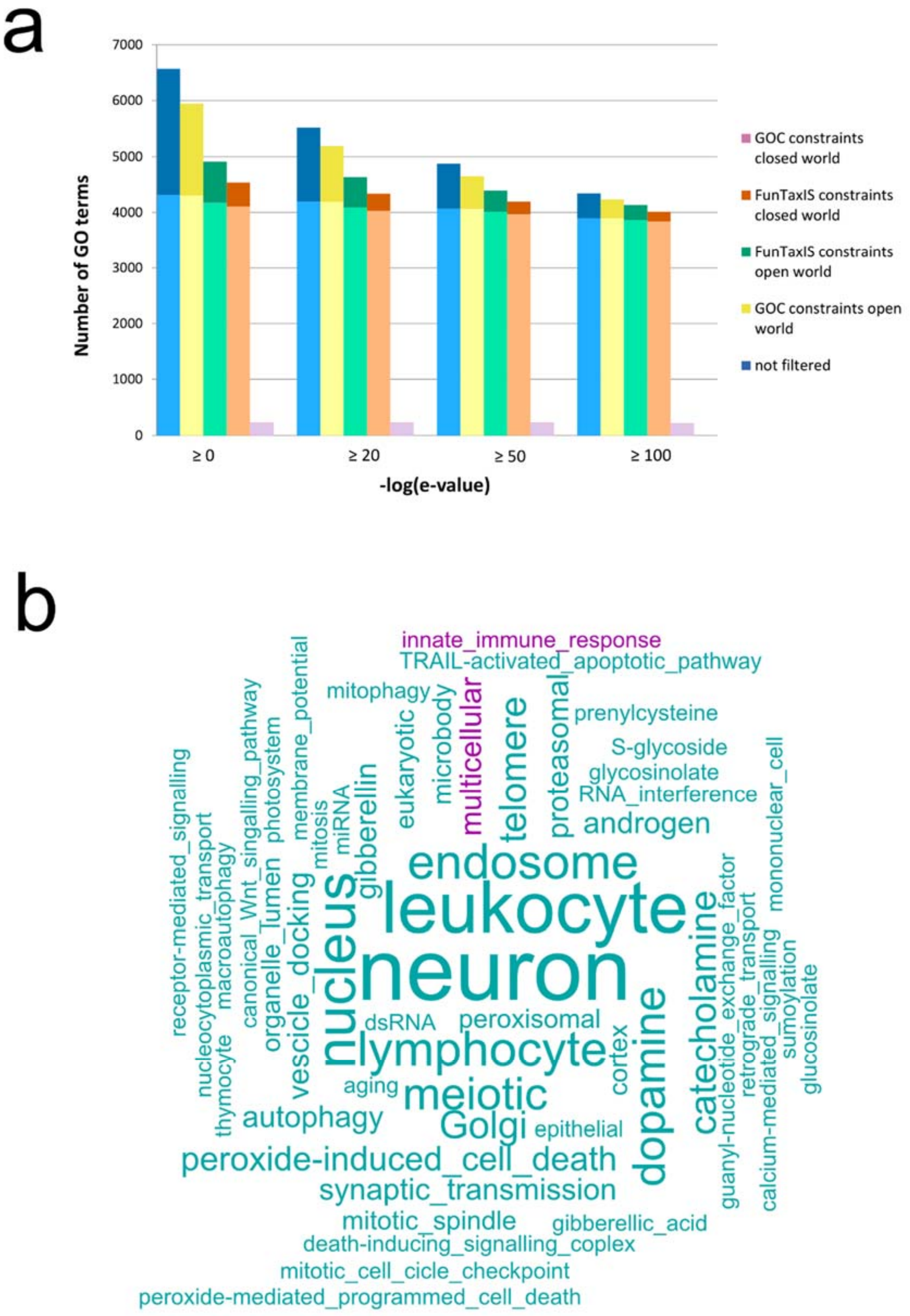

Figure S5.3: GO-centric evaluation of the impact of taxon constraints on *E. coli* annotation by sequence-based functional transfer.

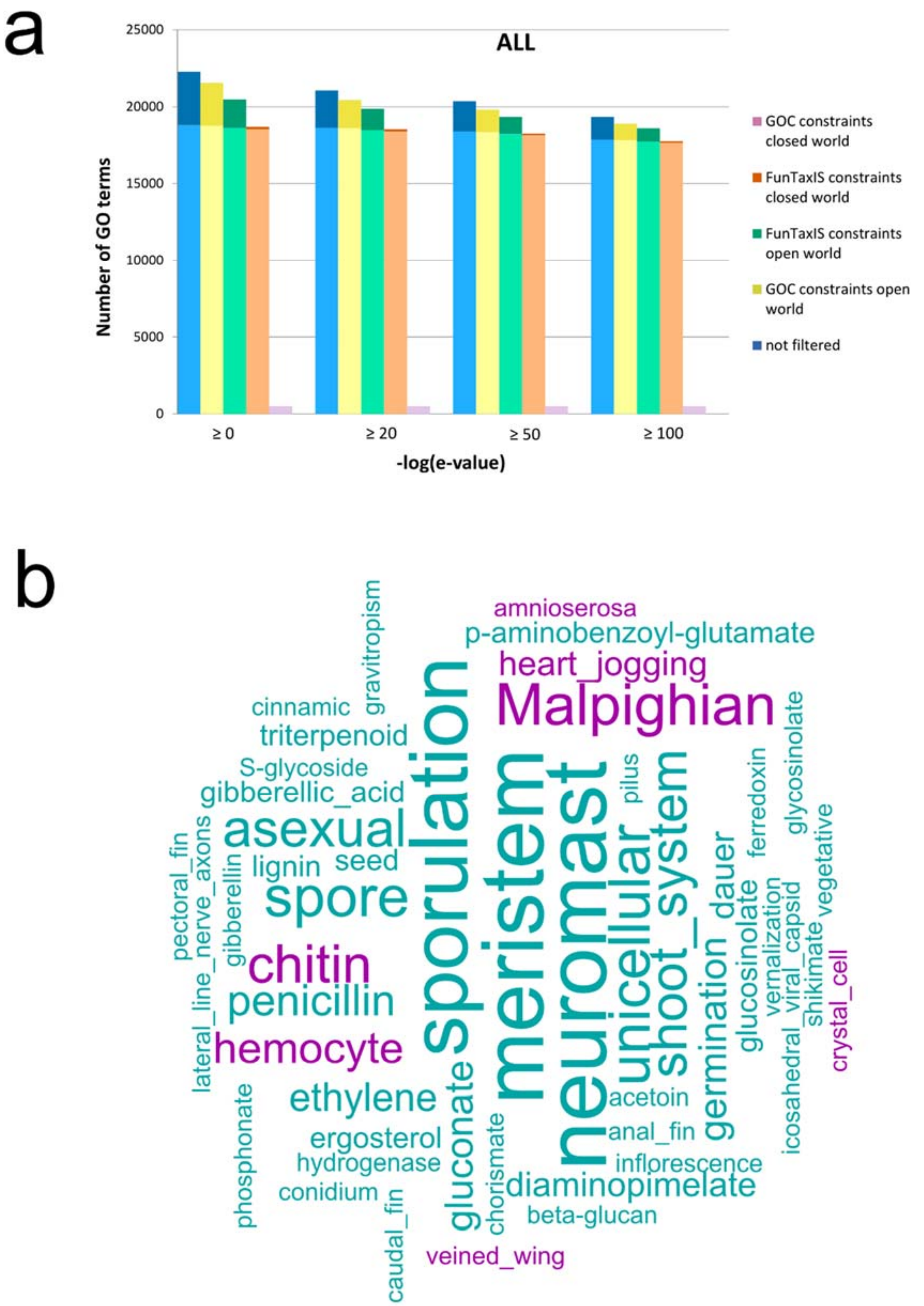

Figure S5.4: GO-centric evaluation of the impact of taxon constraints on *H. sapiens* annotation by sequence-based functional transfer.

S6. The origin of taxon-incompatible GO terms

S6.1 Functional taxonomic consistency vs. alignment significance

To investigate the potential presence of a dependency between the probability for a GO term of being discarded due to the application of a taxon constraint and the significance of the BLAST hit from which it is derived, we plotted the amount of GO terms surviving or not surviving the taxon constraints filtering and their corresponding e-values. The histograms (Figure S6.1) show, for the yeast proteome, the amounts of GO terms coming from BLAST hits that survive (kept) and do not survive (discarded) the filtering step based on taxon constraints. They are divided in bins of log-transformed e-values, which represent the significance of the hit. The bin “<= 300” contains e-values equal to zero. The results show that there is no difference in the e-value distributions of filtered *vs*. not filtered GO terms, suggesting that the significance of pairwise alignments is not a reliable indicator of taxon compatibility of annotations.

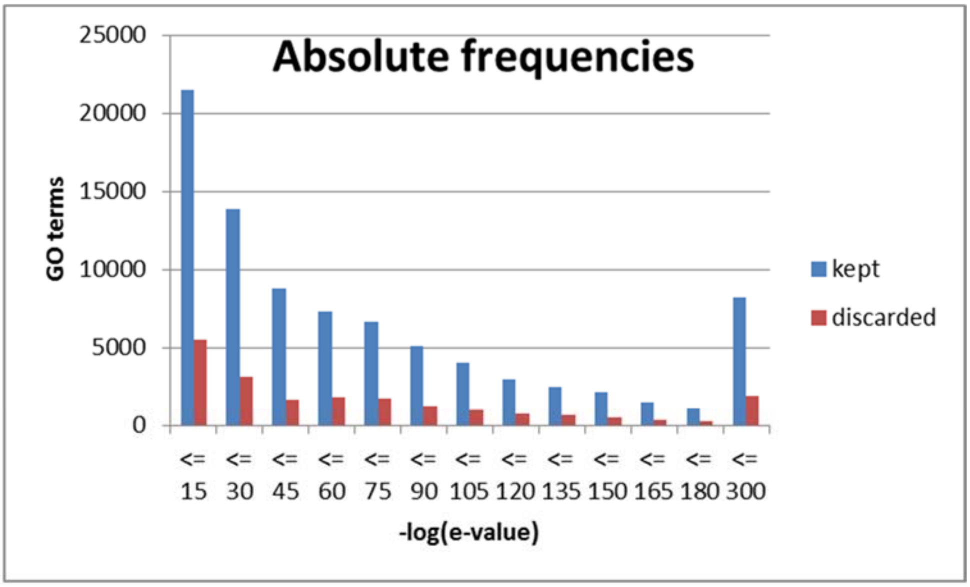

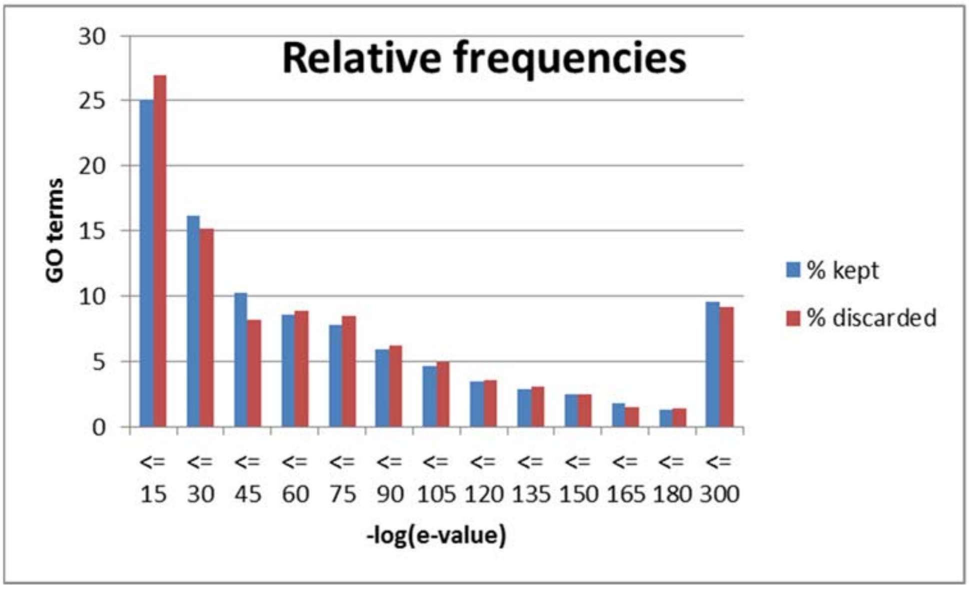

Figure S6.1: GO terms coming from BLAST hits of yeast proteins that survive (kept) and do not survive (discarded) the filtering step based on taxon constraints.

References

1. Peng, J, J Chen, Y Wang. (2013) Identifying cross-category relations in gene ontology and constructing genome-specific term association networks. BMC Bioinformatics, 14, Suppl 2:S15.
